# Loading of Oregano Oil in Natural Nanogel and Preliminary Studies on Its Antiviral Activity on Betacoronavirus 1

**DOI:** 10.3390/molecules30091939

**Published:** 2025-04-27

**Authors:** Lyubomira Radeva, Maya M. Zaharieva, Sevda Naydenska, Pelagia Foka, Erini Karamichali, Efthymia Ioanna Koufogeorgou, Urania Georgopoulou, Stanislav Philipov, Alexander Kroumov, Hristo Najdenski, Ivanka Spassova, Daniela Kovacheva, Krassimira Yoncheva

**Affiliations:** 1 Faculty of Pharmacy, Medical University of Sofia, 2 Dunav Str., 1000 Sofia, Bulgaria; l.radeva@pharmfac.mu-sofia.bg; 2 The Stephan Angeloff Institute of Microbiology, Bulgarian Academy of Sciences, 1113 Sofia, Bulgaria; zaharieva26@yahoo.com (M.M.Z.); adkrumov@gmail.com (A.K.); hnajdenski@gmail.com (H.N.); 3 Faculty of Medicine, Alexandrovska Hospital, Medical University of Sofia, 1431 Sofia, Bulgaria; sevda.naydenska@abv.bg; 4 Department of Microbiology, Laboratory of Molecular Virology, Hellenic Pasteur Institute, 11521 Athens, Greece; pfoka@pasteur.gr (P.F.); uraniag@pasteur.gr (U.G.); 5 Department of Human Anatomy, Histology, General and Clinical Pathology and Forensic Medicine, Faculty of Medicine, University Hospital Lozenetz, Sofia University “St. Kliment Ohridski”, 2 Kozyak Str., 1407 Sofia, Bulgaria; stanislav_philipov@abv.bg; 6 Institute of General and Inorganic Chemistry, Bulgarian Academy of Sciences, 1113 Sofia, Bulgaria; ispasova@svr.igic.bas.bg (I.S.); didka@svr.igic.bas.bg (D.K.)

**Keywords:** oregano oil, chitosan, albumin, nanogel, antiviral activity, Betacoronavirus 1

## Abstract

Oregano oil was successfully encapsulated into chitosan–albumin nanogel via emulsification and electrostatic gelation. The system was characterized with a mean diameter around 26 nm, narrow size distribution (PDI = 0.242) and approximately 40% encapsulation efficiency. The incorporation of the oil into the nanogel was confirmed by XRD and FTIR analyses, and the dissolution of the oil was enhanced after the encapsulation. Furthermore, the treatment of Betacoronavirus 1 infected bovine kidney MDBK cells with the oregano oil-loaded nanogel (25 µg/mL) showed more than 50% protection against the infection, as compared to the non-treated virus infected control. The cytopathic effect (CPE) of the virus was inhibited in a concentration-dependent manner. The system inhibited the virus replication, resulting in a decrease of the viral particles by more than half, as shown by the cytotoxicity and CPE assays. The virus titer in treated and non-treated samples was determined by digital droplet PCR and revealed Δ3 log diminishment of the virus particles in samples treated with 25 µg/mL encapsulated oregano oil. This study is a basis for further investigations on the pharmacodynamics of the nanogel and its possible combinations with clinically applied chemotherapeutics.

## 1. Introduction

Currently, the therapeutic administration of essential oils is gaining a high level of interest. For instance, oregano oil (OrO) is a natural product that is being intensively investigated for application in human and veterinary medicines [1,2,3,4], the pharmaceutical and cosmetic industries, and in the food industry as part of packages for prevention of microbial contamination [5,6,7,8,9]. It possesses remarkable effects such as antioxidant, anti-inflammatory, antidiabetic, antibacterial, antifungal and antiviral activities [10]. The antiviral activity of OrO against different viruses, such as human immunodeficiency virus [11], pseudorabies virus [12], murine norovirus [13], and herpes simplex virus [14] has been proved. Essential oils can target different phases during the virus lifecycle, such as virus adsorption, internalization, or genome replication [15]. Gilling et al. demonstrated that oregano oil inactivates murine norovirus within 1 h by acting directly on the viral capsid and subsequently the RNA [13]. Mexican oregano oil and its main component, carvacrol, were found to inhibit different human and animal viruses in vitro, in particular ACVR-HHV-1 and HRSV, whereby the antiviral effect was most pronounced when viruses or cells were treated before inoculation [16]. Furthermore, since the N-terminal and C-terminal RNA binding domains of SARS-CoV-2 nucleocapsid protein are attractive therapeutic drug targets, the potential of oregano leaf extract to bind these domains has been investigated [17]. In silico studies revealed that the main compounds in the oil, namely carvacrol and thymol, can show moderate activity as G-protein coupled receptors ligands, ion channel moderators, nuclear receptor ligands, protease, kinase and enzyme inhibitors. Furthermore, the affinity of these compounds, especially carvacrol, toward SARS-CoV-2 N-terminal nucleocapsid protein was greater than that of the antiviral drug remdesivir [17]. Oregano oil was found to be effective against neonatal diarrhea syndrome in calves caused by bacterial pathogens or viral infections, including bovine coronavirus [18]. Daily administration of 12.5 mg/kg body weight once per day up to 10 days was enough to diminish the severity of the condition. It is also proven that oregano oil can destroy lipid-encapsulated viruses such as respiratory syncytial virus [19]. Recently, it was observed that carvacrol possesses significant binding affinity to the SARS-CoV-2 papain-like protease [20].

However, the components in oregano oil possess extremely low aqueous solubility, which makes its application difficult and compromises its effects. Loading the oil into nanoparticles is an appropriate strategy to improve its administration. For instance, the antibacterial, antifungal or antioxidant activities of the oil have been enhanced via encapsulation in chitosan–alginate nanoparticles [21], chitosan nanoparticles [22], polyvinyl alcohol–chitosan nanoparticles [23] and poly(ε-caprolactone) nanoparticles [24]. Nanogels are considered alternative nanosized drug delivery systems that are characterized by many advantages. They can be prepared from amphiphilic natural polymers, which makes them capable of improving the biopharmaceutical characteristics of hydrophobic substances. For example, the solubility of *Zingiber officinale* essential oil was improved by encapsulation in chitosan nanoparticles [25]. *Lippia sidoides* essential oil was also successfully encapsulated in biopolymeric sodium alginate/cashew gum nanoparticles, which provided 45–95% release of the oil in 30–50 h [26]. The solubility of the essential oil from orange peel was significantly increased (achieving 93.5%) upon encapsulation in nanocapsules based on gum arabic and maltodextrin [27]. Moreover, the nanogel particles are characterized by biodegradability and biocompatibility [28], high stability, and the ability to protect the loaded active ingredients from internal or external factors [29,30].

Thus, in view of the above evidence, we developed an oregano oil-loaded nanogel (OrO-NG) based on the natural polymers chitosan and albumin. The nanodelivery system was tested for antiviral activity on Betacoronavirus 1 for the first time, in order to evaluate its pharmacological potential for further development as a candidate for antiviral therapy.

## 2. Results and Discussion

### 2.1. Encapsulation of Oregano Oil in the Nanogel and Characterization of the System

**Figure 1:**
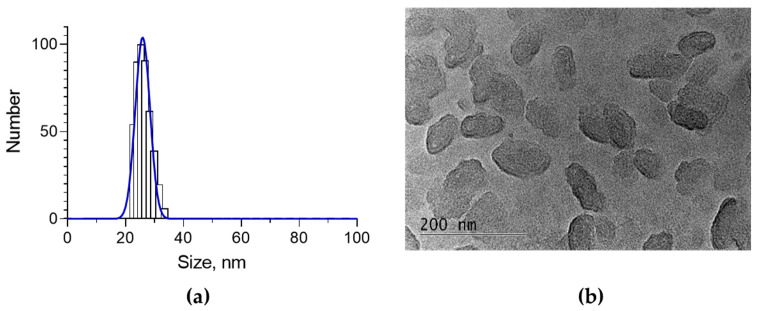
Histogram of the average size (according to DLS analysis) (**a**) and TEM (**b**) of the oregano oil-loaded nanogel.

Incorporation of a hydrophobic agent into nanogel could be a challenge due to the hydrophilic nature of the nanogel. In our study, encapsulation of oregano oil in chitosan–albumin nanogel was performed by modification of the typical gelation method. Specifically, first, the organic solution of the oil was emulsified in an aqueous solution of albumin, and after that, electrostatic gelation was initiated by addition of chitosan solution. According to this procedure, the incorporation of oregano oil into the chitosan–albumin nanogel approximated 39.2% encapsulation efficiency. This value is higher than previously reported results, where the encapsulation efficiency of the oil into chitosan nanoparticles was between 5.45 and 24.72% [31]. However, there are studies reporting higher encapsulation efficiency than that obtained in the current study, for instance, approximately 83% in chitosan nanoparticles [32] and 50% in chitosan–alginate nanoparticles [21]. This is probably due to the high hydrophilicity of our nanogel system. Physicochemical characterization revealed that the oregano oil-loaded nanogel possessed a small mean diameter of 26 nm, and a narrow size distribution, with 0.242 polydispersity index, and positive zeta-potential (20.6 mV) (Figure 1a, Table 1). Transmission electron microscopy (TEM) showed that the nanoparticles possessed an oval shape (Figure 1b) and a larger size compared to the data from DLS analysis. The most probable reason for this difference is the non-spherical shape of the particles, which corresponds to the smaller hydrodynamic diameter measured by the DLS method [33]. According to some studies, the small size and the irregular shape would provide a larger surface area for cellular contact and improve the antiviral effect [34]. Furthermore, the positive charge of the particles could further enable interactions with the negatively charged cell membrane and additionally enhance the effects.

**Figure 2:**
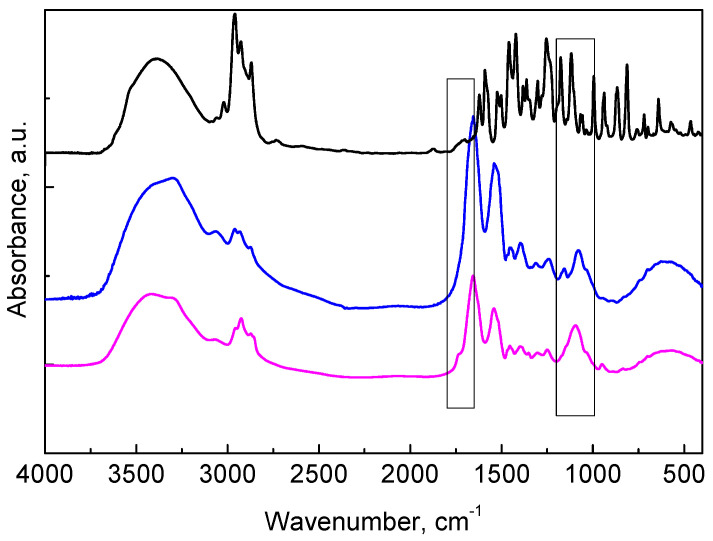
FTIR spectra of oregano oil (black), empty chitosan–albumin nanogel (blue) and oregano oil-loaded chitosan–albumin nanogel (magenta) registered in KBr pellets.

The FTIR analysis revealed that, except for the common O–H, and C–H bands (3500–3200 cm^−1^ and ~2900 cm^−1^, respectively), in the spectrum of pure oregano oil, characteristic bands indicated the presence of carvacrol and thymol in the fingerprint region 900–1200 cm^−1^ (Figure 2). Along with the characteristic chitosan and albumin peaks, the presence of peaks at 1760 cm^−1^ (C=O stretch) and 1122 cm^−1^ (C–O stretch) suggested the presence of phenols (carvacrol and thymol in oregano oil) in the FTIR of the loaded sample. Since carvacrol and thymol are the main compounds in oregano oil, this indicated the successful entrapment of oregano oil into the nanogel.

**Figure 3:**
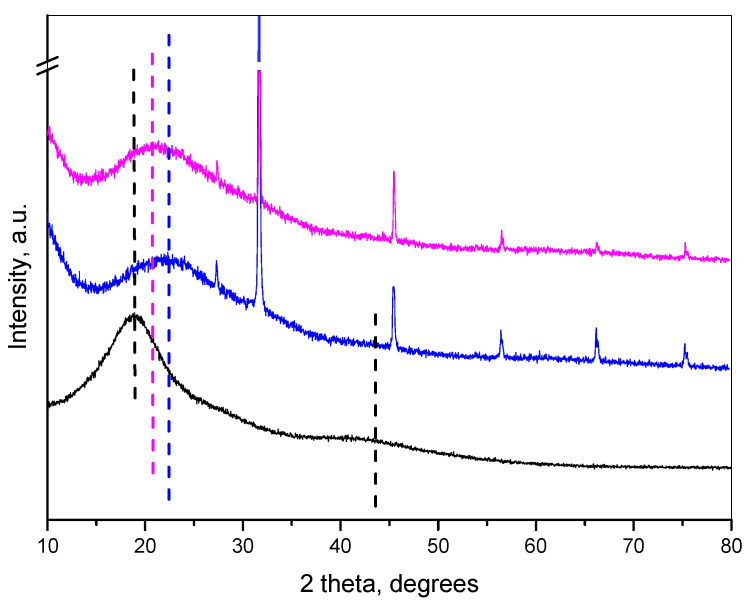
XRD pattern of oregano oil (black), empty chitosan–albumin nanogel (blue) and oregano oil-loaded nanogel (magenta).

The X-ray diffraction pattern (XRD) of the oregano oil showed two diffraction humps, at 18° 2θ and at 42° 2θ (Figure 3). The empty nanogel comprised an amorphous hump centered at 23° 2θ. The pattern of oil-loaded nanoparticles showed a maximum of the broad hump at 21° 2θ. This maximum is situated between the maxima of the pure oregano oil and the chitosan–albumin nanogel. The shift of the maximum of the pattern of the oregano oil toward higher 2θ showed the decrease of the interatomic distances in the loaded nanogel, suggesting densification of the amorphous network compared to the pure oil. At the same time, the oil loading caused a change in the chitosan–albumin nanogel structure. Thus, the changes in the XRD patterns indicated successful loading. The sharp peaks presented in the patterns of the empty nanogel and the loaded sample are due to the presence of NaCl originating from the preparation procedure.

**Figure 4:**
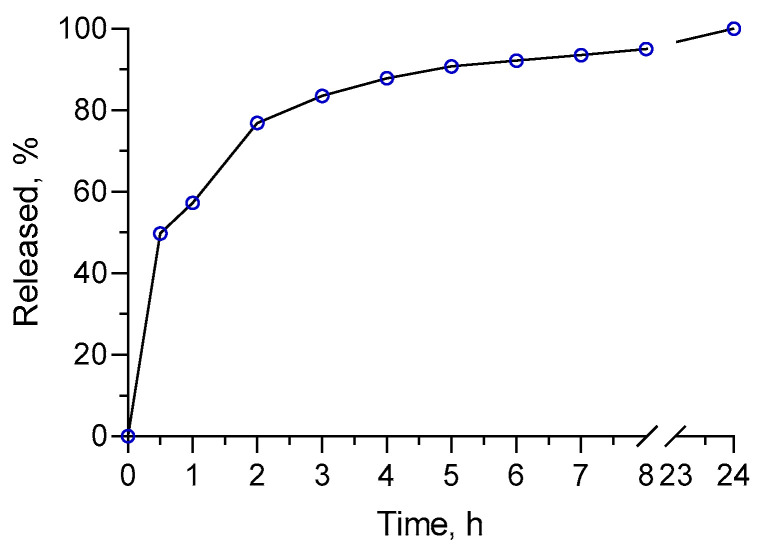
In vitro release profile of oregano oil from the developed nanogel in phosphate buffer medium (pH = 7.4).

The in vitro dissolution test showed a burst release for the first hour, namely, approximately 60% of the oil was found in the acceptor media (Figure 4). Then, a sustained release of the oil was observed for 24 h. This is probably due to the fact that a fraction of the oil was on the surface of the nanoparticles or close to it. Similar results were observed with chitosan nanoparticles, that can be explained by the small size of the nanoparticles, leading to a greater surface-to-volume ratio [31]. Furthermore, the different mechanisms of release, such as surface erosion, disintegration, diffusion, and swelling, could be responsible for this biphasic manner [31]. More importantly, approximately 100% of the oil was dissolved for 24 h. This confirms the ability of the nanogel system to improve its biopharmaceutical behavior.

### 2.2. Studies of the Antiviral Activity of the Loaded Nanoparticles

There are different hypotheses explaining the mechanism of action of essential oils against viruses [13]. For instance, experiments with manuka oil, star anise oil, hyssop oil, thyme oil and ginger oil against herpes simplex viruses suggested that the mechanism is related to direct binding to the virus and likely inhibition of virus adsorption to host cells [14,35,36]. The antiviral effect of tea tree oil or a blend of wild orange, clove, cinnamon, eucalyptus, and rosemary oil was associated with prevention of the adsorption of influenza virus to host cells [37,38]. Furthermore, disruption of the envelope of herpes simplex virus type 1 by carvacrol and thymol was reported by Lai et al. [39]. Gilling et al. found that the capsid of murine norovirus was partially degraded by carvacrol after a brief exposure. Moreover, they suggested that the active substances in oregano oil could bind to the capsid, block epitopes required for virus adsorption to the host cells, or cause a conformational change [13]. Also, according to the literature, carvacrol could directly interact with bacterial cell walls or membranes [40,41,42].

**Figure 5:**
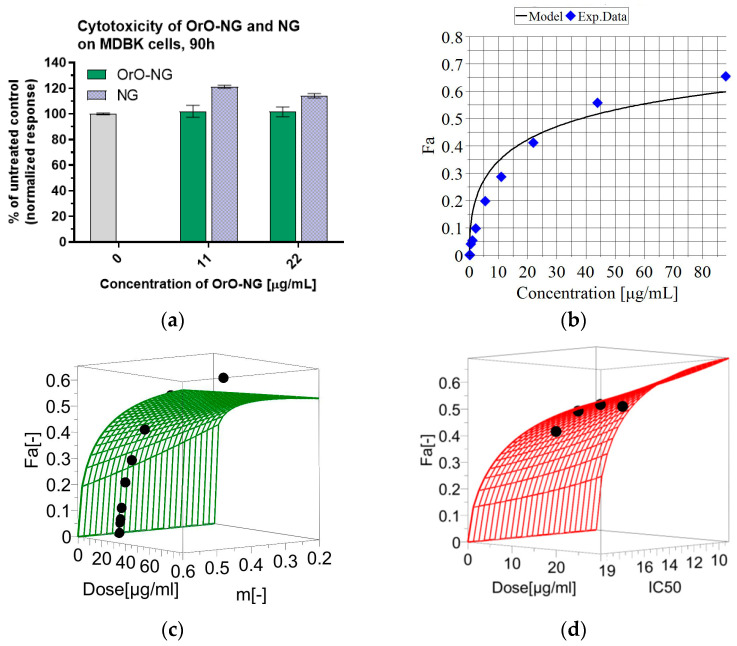
Cytotoxicity of OrO-NG on MDBK cells after 90 h of incubation: (**a**) in vitro cytotoxicity of OrO-NG and NG on MDBK cells after 90 h of incubation—the grey column represents the untreated control which is the same for OrO-NG and NG; (**b**) dose-response curve used for calculation of the median inhibitory concentration (IC_50_) of OrO-NP on MDBK cells after 90 h of incubation presented in Table 2; (**c**) response surface analysis (RSA) for IC_50_ = const; (**d**) RSA for m = const. Legend: OrO-NG—oregano oil-loaded nanogel; NG—empty nanogel; Fa—cytotoxic effect; Exp. Data—experimental data; m—hillslope.

The cytotoxicity of OrO-NG on MDBK cells was tested for 90 h and the mean inhibitory concentration (IC_50_) was calculated (Figure 5a,b). The response surface analysis, which proves the reliability of the mathematical model for calculation of IC_50_, is presented in the graphs in Figure 5c,d. The absolute values of the IC_50_ and the parameters needed for calculation of the model are given in Table 2. The minimal non-toxic concentration (percentage vital cells according to ISO 10993-5 [43]) needed for the antiviral experiments was calculated based on the same model used for determination of IC_50_ and is given in Table 2, and a value of 20 µg/mL was determined. However, for the concentrations used, antiviral experiments were increased up to 30 µg/mL, based on literature data indicating that some active compounds in OrO possesses binding affinity to the virus capsid [13].

**Figure 6:**
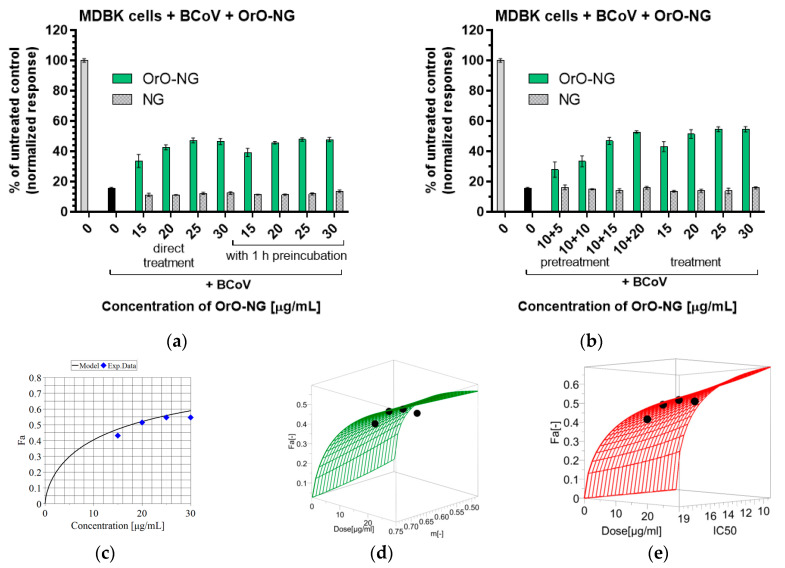
Antiviral activity of OrO-NG on bovine coronavirus after 90 h of treatment: (**a**) direct treatment with or without preincubation—MDBK cells, treated with BCoCV as follows: adding of the virus and OrO-NG or NG together to the cell culture without or after 1 h preincubation of the virus with OrO-NG or NG, the grey column represents the untreated control and the black column represents cells incubated with BCoV; (**b**) pretreatment—incubation of the cultures with 10 µg/mL OrO-NG or NG before inoculation with the virus and adding of 5, 10, 15 or 20 µg/mL OrO-NG or NG after removing the virus; treatment—incubation of the cells with BCoV followed by treatment with OrO-NG or the corresponding concentrations of NG, the grey column represents the untreated control and the black column represents cells incubated with BCoV; (**c**) Antiviral activity of OrO-NG on BCoV—survival curve; (**d**) response surface analysis based on the applied concentration (dose) and the hillslope for IC_50_ = const; (**e**)—response surface analysis based on the dose and IC_50_ value for m = const. Legend: OrO-NG—oregano oil-loaded nanogel; NG—empty nanogel; Fa—cytotoxic effect; Exp. Data—experimental data; m—hillslope.

Thereafter, MDBK cells were treated with concentrations of OrO-NG of up to 30 µg/mL and the equivalent concentration of empty NG before (pretreatment) or after (treatment) adding the virus to the cell culture, or together with the virus (direct treatment). In the case of pretreatment, the cells were preincubated with a low concentration of OrO-NG, infected with the virus and treated again with the loaded nanogel. This approach was least effective. Direct treatment or treatment were equally efficient and did not show significant differences. Both approaches were more effective than pretreatment. A concentration of 25 µg/mL OrO-NG achieved the maximal protection (approx. 50%) on treated cells against the virus CPE as compared to the non-treated virus infected control (Figure 6). The median effective concentration of OrO-NG was 17.75 µg/mL (Table 2), which determines a selectivity index of 2.17. The decreased toxicity of the oil on the infected cells is probably due to engagement of the active centers of the compounds with fragments from the virus.

**Figure 7:**
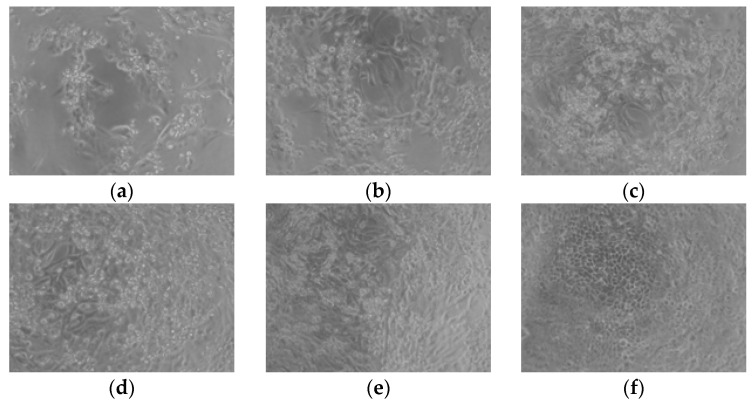
Cytopathic effect of bovine coronavirus on MDBK cells with and without OrO-NG treatment—200× magnification. Legend: (**a**) BcoV, bovine coronavirus; (**b**) 15 μg/mL OrO-NG; (**c**) 20 μg/mL OrO-NG; (**d**) 25 μg/mL OrO-NG; (**e**) 30 μg/mL OrO-NG; (**f**) Control cells without virus and treatment.

The cytopathic effect of BCoV on MDBK cells after treatment with different concentrations of OrO-NG is presented on Figure 7. As shown in changes in cellular morphology on the micrographs, there is a concentration-dependent inhibition effect on the CPE that is most pronounced at 25 and 30 µg/mL. Cell rounding, detachment, and clumping of adherent cells were observed in samples treated with 15 µg/mL of OrO-NG. The cell density in these samples was uneven, and small number of cells were characterized by a regular morphology and proportional cytoskeletal changes. In cultures treated with 25 and 30 µg/mL of OrO-NG, the morphological signs corresponded to lower virus replication levels, whereby cells showed a picture of proliferation and the cell morphology was closer to that of the untreated control. The cell density increased on a concentration-dependent manner and approached that of untreated cells.

**Figure 8:**
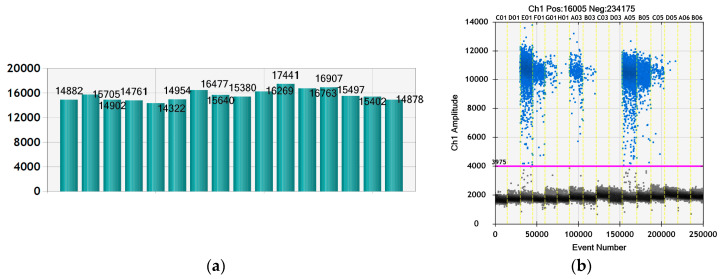
Digital droplet PCR for enumeration of viral particles in OrO-NG treated samples and the virus control—dilution of the cDNA. Legend: (**a**) Number of generated droplets per sample; (**b**) Histogram of the positive and negative droplets in each sample—the fluorescent positive droplets (blue) are separated on the histogram from the non-fluorescent negative droplets (grey) through the threshold line (pink color) generated by the data analysis with the QuantaSoft^®^ Software of the ddPCR device.

In order to quantify the viral particles in treated and non-treated samples for the planned experiment, we performed a ddPCR as described previously [44]. The viral particles in the stock were determined in the above-cited publication and according to the ddPCR data the initial virus titer was 2.24 × 10^10^/mL [44]. The number of generated droplets varied between 14,322 and 17,441 (Figure 8a), which is a marker for the reliability of the reaction, i.e., enough droplets were generated to ensure the statistical evaluation of the data and the calculation of the positive and negative events. The histogram in Figure 8b shows the positive fluorescent droplets and the negative non-fluorescent droplets. The calculation of the cDNA concentration in each sample was performed with QuantaSoft^®^ software of the ddPCR device and is presented in Table 3.

Treatment of BCoV infected MDBK cells with 25 µg/mL OrO-NG led to diminishment of the virus titer by half, i.e., with more than Δ3 log (5 × 10^3^), which corresponds to the data from the cytotoxicity assay and the microscopy. Finally, the number of virus particles per sample was as follows: 7.85 × 10^6^/100 µL in infected cells without treatment, 7.03 × 10^6^/100 µL in infected cells incubated with NG and 2.32 × 10^3^/100 µL in samples treated with 25 µg/mL OrO-NG. As shown in the results, the empty nanogel NG did not produce significant diminishment of the viral particles as compared to the BCoV control in difference to the oil-loaded OrO-NG.

## 3. Materials and Methods

### 3.1. Materials

Chitosan (Mv 110,000–150,000), bovine serum albumin (fraction V) and Tween 80 were purchased from Sigma Chemical Co. (Merck, Darmstadt, Germany). *Origanum vulgare* was cultivated in the area of Panagyurishte (a mountainous region in south Bulgaria) and the essential oil was obtained by distillation method via Clevenger apparatus [21]. The active components of the essential oil, namely 39.44% o-cymene/m-cymene, 29.80% carvacrol, 20.82% terpinolene, 4.05% γ-terpinene, 3.23% thymol, 1.08% aromadendrene and 1.05% trans-β-ocimene/α-pinene, were determined via thin-layer chromatography and gas chromatography–mass spectrometry (GC–MS) in a previously published phytochemical analysis [21]. The culture media, sera and buffers were purchased from Capricorn Scientific, Ebsdorfergrund, Germany: Minimal Essential Medium (MEM) with Earle’s Salts (#MEM-A), fetal bovine serum (#FBS-HI-12A), pen/strep 100× (#PS-B), stable L-glutamine, non-essential amino acids (NEAA) and Accutase^®^ (#ACC-1B). The dye 3-(4,5-dimethylthiazolyl-2)-2,5-diphenyltetrazolium bromide (MTT, #M2128-1G), Dulbecco’s phosphate-buffered saline (PBS, #D8537) and sodium pyruvate (#S8636) were purchased from Merck (Sigma-Aldrich, Steinheim, Germany).

### 3.2. Preparation of Oregano Oil-Loaded Nanogel

The oregano oil loaded nanogel was prepared via emulsification and further electrostatic gelation [45]. Briefly, 10 mg Tween 80 was added to 5 mL 0.5% aqueous solution of albumin. Then, 10 mg oregano oil, previously dissolved in methylene chloride (0.115 mL), was emulsified in the solution of albumin under sonication (20 Hz, 1 min, Bandelin Sonopuls, Berlin, Germany). Chitosan solution in acidic buffer (pH = 1.2, 1 mL, 0.5%) was dripped in and the dispersion was stirred for 90 min (700 rpm). The pH of the dispersion was adjusted to approximately 4.6 by adding NaOH. Then, the sample was heated for 20 min at 78 °C and stirred for 3 h. The formulation was filtered (0.2 µm) and the concentration of the oil was determined spectrophotometrically at λ = 275 nm (Thermo Fisher Scientific, Waltham, MA, USA) [46]. For the calculation of the concentration a standard curve of the oil in methanol in 5.31–63.8 µg/mL range (r > 0.998) was used.

The encapsulation efficiency (EE) and drug loading (DL) were determined according to the following equations:

EE = (Initial amount of oregano oil − Non-loaded oregano oil)/Initial amount of oregano oil(1)

LD = (Initial amount of oregano oil − Non-loaded oregano oil)/Volume of loaded nanogel dispersion(2)

### 3.3. Characterization of the Loaded Nanogel

The size and the polydispersity index of the loaded nanogel were determined via photon correlation spectroscopy at a scattering angle of 90° (Zetasizer NanoBrook 90Plus PALS, Brookhaven Instruments Corporation, Holtsville, NY, USA). The phase analysis light scattering (PALS) method at a scattering angle of 15° was carried out for the determination of zeta potential of the systems. The morphology of the nanogel was investigated by transmission electron microscopy (HR STEM JEOL JEM 2100, Tokyo, Japan).

FTIR analysis was conducted using a Nicolet iS5 FTIR spectrometer (Thermo Fisher Scientific, Waltham, MA, USA) by accumulating 64 scans at a spectral resolution of 2 cm^−1^.

A Bruker D8 Advance diffractometer (Bruker Corporation, Billerica, MA, USA) with Cu Kα radiation and a LynxEye detector in the 5–80° 2θ range with a step of 0.02° were used for recording the powder X-ray diffraction patterns of free oregano oil, empty and loaded nanoparticles.

### 3.4. In Vitro Release Study

The in vitro release test was conducted via a dialysis method in a phosphate buffer with pH value of 7.4. Briefly, 4 mL nanogel dispersion was placed into a membrane (10,000 MWCO, Spectrum Labs, San Francisco, CA, USA). It was then submerged in 40 mL of release medium and gently shaken at 37 °C (IKA Labortechnik HS-B20, Staufen, Germany). At predetermined time intervals, samples of 3 mL were taken and the same amount of fresh buffer was returned. The concentration of the released oregano oil in the samples was determined spectrophotometrically, as described above.

### 3.5. Cultivation of MDBK Cells and Propagation of Betacoronavirus 1

The Madin–Darby bovine kidney cell line MDBK (NBL-1, #600396, CLS Cell Lines Service GmbH, Eppelheim, Germany) was used for propagation of the bovine coronavirus strain “S379 Riems” (022V-04370 Betacoronavirus 1, FLI, WOAH Collaborating Centre for Zoonoses in Europe, Greifswald—Insel Riems, Germany) and evaluation of the antiviral activity of the encapsulated oregano oil. The recommendations of the biobank were followed by maintaining the cell culture. The culture medium consisted of MEM, supplemented with 2 mM GlutaMAX™ (Thermo Fisher Scientific), 0.1 mM non-essential amino acids, 1 mM sodium pyruvate and 10% (*v*/*v*) FBS. The cells were cultured in sterile 25 cm^2^ flasks in a CO_2_ incubator (Panasonic MCO-18AC, Panasonic Healthcare Co., Ltd., Oizumi-Machi, Japan) at 37 °C, 5% (*v*/*v*) CO_2_ and humidified atmosphere. After reaching 90% confluence, the cell cultures were subcultured using the enzyme solution Accutase^®^ (Capricorn Scientific GmbH, Ebsdorfergrund, Germany) every 3 d at a ratio 1:4. The titer of the virus stock used for all experiments was 2.24 × 10^10^/mL, as previously published [44].

### 3.6. In Vitro Cytotoxicity of Encapsulated Oregano Oil on MDBK Cells

The in vitro cytotoxicity of OrO-NG and NG was determined in accordance or with modifications according to ISO 10993-5, Annex C [43,47] in order to determine the maximal non-toxic concentrations for MDBK cells. Briefly, the cells were plated at a density of 0.135 ×10^6^ cells/mL in 96-well plates in a volume 100 µL/well. The plates were incubated for 24 h to allow the cells to enter the log-phase of the growth curve. OrO-NG was prepared in 10 serial two-fold decreasing dilutions in PBS before being added to the cells. The concentrations ranged varied from 0.086 to 44 µg/mL in 10 concentrations. After 90 h of incubation in a CO_2_ incubation, the cell viability was measured according to Annex C of the above-cited standard. The absorbance was measured at λ = 550 nm (reference filter at 690 nm) on a microplate reader ELx800 (BioTek Instruments, Inc., Winooski, VT, USA). The IC_50_ value was calculated in MAPLE^®^ mathematical software (Version 15, Maplesoft, a division of Waterloo Maple Inc., Waterloo, Ontario).

### 3.7. Determination of Antiviral Activity

The antiviral activity of OrO-NG and NG was determined via four different approaches—direct inactivation of the viral particles before adding the viral suspension to the MDBK cells, pretreatment of the cell cultures with OrO-NG or NG followed by a treatment, and treatment after virus contamination and removing the virus from the cells. The cytopathic effect (CPE) was evaluated microscopically regarding the CPE and confirmed biochemically with the MTT-dye reduction assay according to ISO 10993-5/2009, Annex C with some modifications [43,47]. Briefly, 0.135 × 10^6^ cells/mL cells were plated in 96-well plates in a volume of 100 µL/well. After 24 h the medium was aspirated and replaced with 100 µL/well viral inoculum with or without OrO-NG or NG, as described in the Results section. The viral inoculum was prepared in 2% (*v*/*v*) FBS medium MEM at MOI = 1. The plates were left in a 5% CO_2_ incubator (37 °C, humidified atmosphere) for 3 h. Thereafter, the supernatant in all wells was replaced by regular 10% (*v*/*v*) FBS medium MEM. In the case of the pretreatment approach, the samples were additionally treated with OrO-NG or NG according to the schema presented in the Results section. The samples were further incubated for 4 d, evaluated daily for signs of CPE under an inverted microscope and documented with micrographs. The cell survival was determined with the MTT-dye reduction assay, as described above. The maximal effective concentration 50% (EC_50_) achieving 50% protection of cells from the virus-induced death was calculated using a non-linear mathematical model in MAPLE^®^ software. The selectivity index (SI) was calculated based on the results after treatment as the ratio of the IC_50_ value in MDBK cells and EC_50_ regarding the BCoV inactivation [48].

### 3.8. Mathematical Model for Calculation of Median Inhibitory and Maximal Non-Toxic Concentration

MAPLE^®^ mathematical software was applied for calculation of the cytotoxic and antiviral effects of OrO-NG and NG after developing programs for non-linear modeling and response surface analysis (RSA) of the experimental data points. The mathematical models used are based on the Chou and Talaly theory [49,50]. The weighted least squares statistical criterion as an objective function of the search was applied by coding of the non-linear regression procedure in MAPLE^®^ software of symbolic mathematics. A numerical optimization algorithm was used to minimize the sum of weighted squares and to estimate the best-fitting parameter values. The median-dose model applied for calculation of “*IC*_50_” and “*m*”, was as follows:

(3) FaFu=DoseDmm,

where *F_a_*—affected fraction; *F_u_*—unaffected fraction (1 − *F_a_*) = *F_u_*; *Dose*—applied compound concentration; *D_m_*—median-effect concentration (in our study *D_m_* = *IC*_50_); and *m*—a hillslope of the median-effect plot (for *m* = 1 the curve is hyperbolic; for *m* > 1—sigmoidal; for *m* < 1—negative (flat) sigmoidal).

A response surface analysis (RSA) methodology revealed the predictive power of the model as a function of the parameters “*IC*_50_” and “*m*”. The range of the parameters’ values changes was presented in RSA 3D plots based on the standard deviation of the “*IC*_50_” and “*m*” values. The standard deviation values were taken from the statistical evaluation of the experimental data with GraphPad Prism software (Version 6.00, for Windows, GraphPad Software, La Jolla, CA, USA). The maximal non-toxic concentrations were also calculated with GraphPad Prism with the [log(inhibitor) vs. normalized response − variable slope] model:

(4) $$Y=\frac{100}{1+10^{(log{IC}_{50}-X)\times hillslope}}.$$

### 3.9. Determination of the Bovine Coronavirus Titer with Droplet Digital (ddPCR) After Treatment with OrO-NG or NG

The bovine coronavirus RNA was extracted with the NucleoSpin RNA Virus kit (Marcherey-Nagle GmbH, Düren, Germany). The RNA concentration was measured on a NanoDrop™ Lite spectrophotometer (ThermoFisher Scientific, Waltham, MA, USA). The reverse transcription was performed with PrimeScript^TM^ Reverse Transcriptase (TaKaRa, Kusatsu, Japan). For each sample, four tenfold serial dilutions (from 10^−1^ to 10^−4^) were prepared with the cDNA. The ddPCR reaction was carried out as previously described [44]. The primers/probe sequences (complementary for the nucleocapsid gene of Betacoronavirus 1) were as follows: FP 5′-GGACCCAAGTAGCGATGAG-3′; RP 5′-GACCTTCCTGAGCCTTCAATA-3′ and probe 6-FAM-5′-ATTCCGACTAGGTTTCCGCC-TGG-3′-BHQ1 [51].

### 3.10. Statistical Evaluation

Statistical evaluation of the experimental data was performed in GraphPad Prism software (Version 6.00, for Windows, GraphPad Software, La Jolla, CA, USA). Each sample, including the controls, was presented in triplicate, therefore each experimental point is a mean value ± SD of all three samples. The statistical analysis (comparison between the experimental groups) was carried out with one-way ANOVA, whereby a *p*-value *p* < 0.05 was considered statistically significant.

## 4. Conclusions

The nanogel prepared from the natural polymers chitosan and albumin could be considered an appropriate carrier for the delivery of oregano oil, since it provides an aqueous nanodispersed form of the oil (26 nm) and an improved dissolution rate. Taking into account the good safety profile of this essential oil, characterized by low cytotoxicity in cultured cells, the activity of the loaded nanosystem against Betacoronavirus 1 (50% protection of bovine kidney MDBK cells against the virus infection) suggests further investigations on the pharmacodynamics of the oil-loaded nanogel and its possible combinations with clinically applied chemotherapeutics.

## Figures and Tables

**Table 1:** Physicochemical properties of the empty (NG) and oregano oil-loaded nanogel (OrO-NG).

Sample	Size, nm	PDI	Zeta Potential, mV	EE, %
Empty NG	29.2 ± 5	0.241	35.5	-
OrO-NG	25.9 ± 2.6	0.242	20.6	39.2

**Table 2:** Cytotoxicity and antiviral activity of OrO-NG on MDBK cells.

Parameters	Cytotoxicity	Antiviral Activity
IC_50_, 90 h	38.55 µg/mL	-
100% vital cells	20 µg/mL	-
EC_50_ (treatment)	-	17.75 µg/mL
R	0.990	0.995
m	0.48	0.68
Selectivity index	2.17	

**Table 3:** Virus titer in OrO-NG treated and non-treated samples.

Well	Sample	Copies per ddPCR Reaction	Copies per Sample (100 µL)
C01	Untreated control	0	0.00
D01	Untreated control	0	0.00
E01	BCoV dilution 10^−1^	14,180	7.85 × 10^5^
F01	BCoV dilution 10^−2^	1060	5.86 × 10^4^
G01	BCoV dilution 10^−3^	104	5.75 × 10^3^
H01	BCoV dilution 10^−4^	6.2	3.43 × 10^2^
A03	OrO-NG 25 µg/mL dilution 10^−1^	420	2.32 × 10^3^
B03	OrO-NG 25 µg/mL dilution 10^−2^	28	1.55 × 10^2^
C03	OrO-NG 25 µg/mL dilution 10^−3^	0	0.00
D03	OrO-NG 25 µg/mL dilution 10^−4^	0	0.00
A05	NG 25 µg/mL dilution 10^−1^	12,700	7.03 × 10^5^
B05	NG 25 µg/mL dilution 10^−2^	1232	6.82 × 10^4^
C05	NG 25 µg/mL dilution 10^−3^	132	7.30 × 10^3^
D05	NG 25 µg/mL dilution 10^−4^	6	3.32 × 10^2^
A06	PCR water	0	0.00
B06	PCR water	0	0.00

## Data Availability

Data are contained within the article.
